# Evolving trends in the surgical therapy of patients with endometrial cancer in Germany: analysis of a nationwide registry with special emphasis on perioperative outcomes

**DOI:** 10.1007/s00404-023-07127-0

**Published:** 2023-07-03

**Authors:** Katrin Roth, Klaus Kaier, Peter Stachon, Constantin von zur Mühlen, Peter Jungmann, Juliane Grimm, Maximilian Klar, Ingolf Juhasz-Böss, Florin-Andrei Taran

**Affiliations:** 1 Faculty of Medicine, Department of Obstetrics and Gynecology, University Medical Center Freiburg, University of Freiburg, Hugstetter Str. 55, 79106 Freiburg, Germany; 2 Faculty of Medicine and Medical Center, Institute of Medical Biometry and Statistics, University of Freiburg, Freiburg, Germany; 3 Faculty of Medicine, Department of Cardiology and Angiology I, University Heart Center Freiburg, University of Freiburg, Freiburg, Germany; 4 Faculty of Medicine, Department of Cardiology and Angiology I, Center of Big Data Analysis in Cardiology (CeBAC), Heart Center Freiburg University, University of Freiburg, Freiburg, Germany

**Keywords:** Endometrial cancer, Robotic-assisted laparoscopic surgery, Laparoscopic surgery

## Abstract

**Purpose:**

Endometrial cancer (EC) is the most common gynecological malignancy in women, with increasing incidence in the last decades. Surgical therapy is the mainstay of the initial management. The present study analyzed the evolving trends of surgical therapy in Germany in patients diagnosed with EC recorded in a nationwide registry.

**Methods:**

All patients with the diagnosis of EC undergoing open surgery, laparoscopic surgery, and robotic-assisted laparoscopic surgery between 2007 and 2018 were identified by international classification of diseases (ICD) or specific operational codes (OPS) within the database of the German federal bureau of statistics.

**Results:**

A total of 85,204 patients underwent surgical therapy for EC. Beginning with 2013, minimal-invasive surgical therapy was the leading approach for patients with EC. Open surgery was associated with a higher risk of in-hospital mortality (1.3% vs. 0.2%, *p* < 0.001), of prolonged mechanical ventilation (1.3% vs. 0.2%, *p* < 0.001), and of prolonged hospital stay (13.7 ± 10.2 days vs. 7.2 ± 5.3 days, *p* < 0.001) compared to laparoscopic surgery. A total of 1551 (0.04%) patients undergoing laparoscopic surgery were converted to laparotomy. Procedure costs were highest for laparotomy, followed by robotic-assisted laparoscopy and laparoscopy (8286 ± 7533€ vs. 7083 ± 3893€ vs. 6047 ± 3509€, *p* < 0.001).

**Conclusion:**

The present study revealed that minimal-invasive surgery has increasingly become the standard surgical procedure for patients with EC in Germany. Furthermore, minimal-invasive surgery had superior in-hospital outcomes compared to laparotomy. Moreover, the use of robotic-assisted laparoscopic surgery is increasing, with a comparable in-hospital safety profile to conventional laparoscopy.

## What does this study add to the clinical work

**Table:** 

The present analysis shows that minimal-invasive surgery has increasingly become the standard surgical technique for patients with EC in Germany, with excellent in-hospital outcomes in clinical practice. Robotic-assisted laparoscopy is an emerging surgical procedure in Germany with promising results compared to conventional laparoscopy.	

## Introduction

Endometrial cancer (EC) is the most common gynecological malignancy in Europe and continues to be a major cause of morbidity and mortality [1]. The estimated number of new EC cases in Europe in 2018 was 121 578, accounting for 29 638 deaths. The incidence of EC has been rising steadily, with an aging population and increased obesity [1]. In 1988, the FIGO introduced the concept of surgical staging of EC in order to provide reliable information about the pathological morphology of the primary tumor and lymph node status, as well as the resulting prognosis and possible indication for adjuvant therapy [2, 3]. Over the last decades, minimal-invasive surgical approaches came increasingly into use. Favorable complication rates, shorter hospital stay, and comparable outcomes compared to open surgery turned laparoscopy into the standard surgical approach [4, 5].

A survey conducted by the German Gynecologic Laparoscopy Working Group highlighted the fact that more than 50% of the surgical procedures for EC in Germany in 2012 were carried out by laparoscopy [6]. Laparoscopy has been found to have similar outcomes to laparotomy for early-stage [4, 7–11] EC in terms of survival and complications. The European (European Society of Gynaecological Oncology, European Society of Medical Oncology, European Society for Radiotherapy and Oncology, European Society of Pathology) and German guidelines for the management of patients with EC recommend minimal-invasive surgery as the preferred surgical approach for patients with early-stage disease [1, 12–14]. Current evidence suggests that robotic-assisted surgery may be advantageous in specific patient groups, such as obese patients, where conventional laparoscopic surgery may be more difficult to perform [15].

The main aim of this study was to analyze and describe the evolving trends in the surgical treatment of EC in Germany from 2007 and 2018, with special emphasis on the surgical technique and perioperative outcomes.

## Methods

Since 2005, data on all hospitalizations in Germany are available for scientific use via the Diagnosis-Related Groups statistics collected by the Research Data Center of the Federal Bureau of Statistics (DESTATIS). These hospitalization data, including diagnoses and procedures, are a valuable source of representative nationwide data on the in-hospital treatment of patients. This database represents a virtually complete collection of all hospitalizations in German hospitals that are reimbursed according to the Diagnosis-Related Groups system. From this database, we extracted data on patients with EC [ICD-10 (International classification of diseases, Version 10) C54*] who underwent surgical therapy [Operation and Procedure Codes (OPS) 5–683.0, 5–683.1, or 5–683.2] by laparotomy, laparoscopy, or by robotic-assisted laparoscopy (OPS 5–987). Finally, three groups of patients who underwent surgery for EC were compared: patients who underwent laparotomy, patients who underwent laparoscopy, and patients who underwent robotic-assisted laparoscopy.

Our study did not involve direct access by the investigators to data on individual patients, the Research Data Center solely provided access to summary results. Therefore, approval by an ethics committee and informed consent were not applicable, in accordance with German law. All summary results were anonymized by DESTATIS. In practice, this means that any information allowing the drawing of conclusions about a single patient or a specific hospital was censored by DESTATIS in order to guarantee data protection. Moreover, in order to prevent the possibility to draw conclusions to a specific hospital, the data are verified and situationally censored by DESTATIS.

In-hospital mortality, length of mechanical ventilation during operation and postoperative discharge destination, length of hospital stay, and reimbursement were part of DESTATIS’ main set of variables. Hence, our analysis focused on six different endpoints: in-hospital mortality, mechanical ventilation, discharge destination (home, other hospital or rehabilitation facility), length of hospital stay, and reimbursement. The baseline characteristics were used to calculate the Charlson Comorbidity Index (CCI) in order to assess general patients health status [16].

No imputation for missing values could be conducted due to the absence of codes indicating that data were missing. If the patients electronic health record did not include information on a clinical characteristic, it was assumed that that characteristic was not present. Time trends were calculated using linear regression models. Between-group differences of patient characteristics and in-hospital outcomes were calculated using unpaired t-test and Fisher’s exact test without adjustment for multiple testing. Thus, p-values may not be interpreted as confirmatory but are descriptive in nature. All statistical analyses were performed with Stata 16 (StataCorp, College Station, Texas, USA).

## Results

### Trends of surgical therapy techniques for EC in Germany between 2007 and 2018

**Fig. 1:**
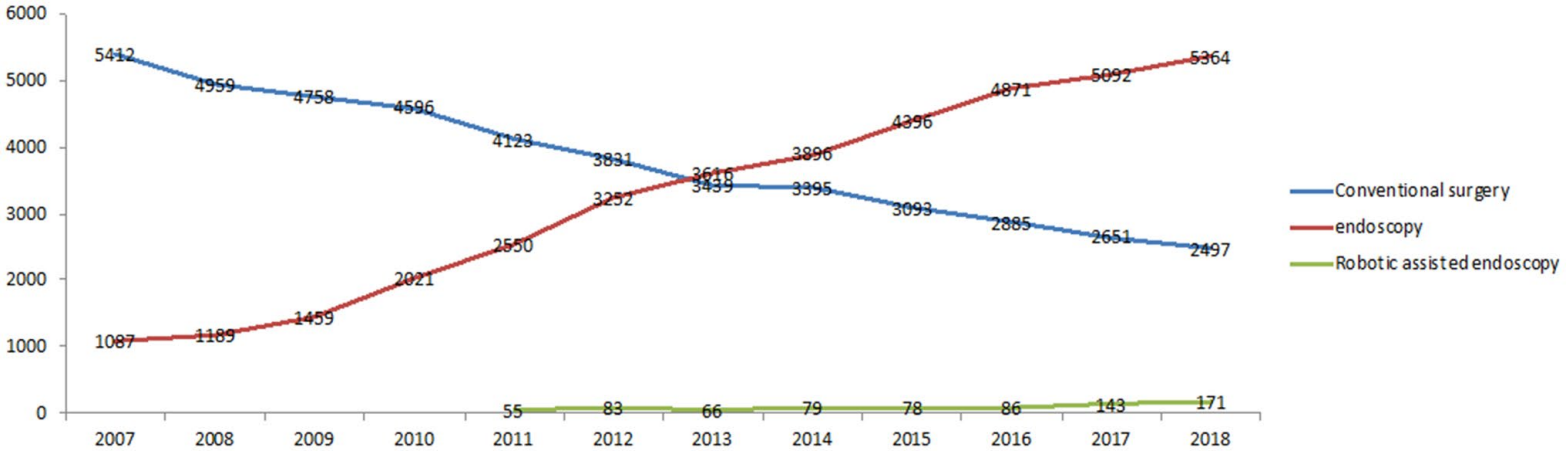
All surgical procedures in patients with endometrial cancer were identified by International classification of diseases (ICD) codes and operational procedures (OPS) codes. The blue line represents the open surgical procedures per year, the red line represents the laparoscopic surgical procedures per year, the green line represents the robotic-assisted surgical procedures per year

Between 2007 and 2018, a total of 85 204 patients underwent surgical therapy for EC in Germany (Fig. 1). While the overall number of surgical procedures for EC increased from 6499 surgeries in 2007 to 8032 surgeries in 2018 (*p* < 0.001), the annual number of laparotomies decreased from 5412 in 2007 to 2497 in 2018 (*p* < 0.001), with the number of minimal-invasive surgical procedures increasing from 1087 in 2007 to 5364 in 2018 (*p* < 0.001) (Fig. 1). In 2011, robotic-assisted laparoscopic procedures were first recorded in the database. The number of robotic-assisted laparoscopic procedures increased from 55 in 2011 to 171 in 2018, but still account to a very low share of all minimal-invasive surgical procedures, 3.2% in 2018 (Fig. 1).

### Baseline characteristics

In comparison to patients with EC undergoing laparotomy, patients who underwent laparoscopic surgery were significantly younger (*p* < 0.001) and were at lower risk of severe outcome and death according to the Charlson Comorbidity Index (CCI) (*p* < 0.001). Among the patients undergoing minimal-invasive surgery, patients undergoing robotic-assisted laparoscopy were significantly younger (*p* < 0.001) and at higher risk of severe outcome and death according to the CCI (*p* < 0.001) (Table 1).

**Table 1:** Baseline characteristics of the patients with a diagnosis of endometrial cancer

Variable	Laparotomy *n* = 45.639 Mean ± SD	Laparoscopy *n* = 38.793 Mean ± SD	Robotic-assisted laparoscopy *n* = 772 Mean ± SD	*p*-value Laparoscopy vs. Robotic-assisted laparoscopy	*p*-value
Age (years)	68.29 ± 11.84	66.24 ± 11.77	63.04 ± 11,40	< 0.001	< 0.001
CCI*	3.33 ± 2.22	2.65 ± 1.41	2.84 ± 1.78	< 0.001	< 0.001

*CCI Charlson Comorbidity Index, *SD*  standard deviation

### In-hospital outcomes laparotomy versus laparoscopic surgery for EC

Patients undergoing laparotomy for EC were associated with a higher risk of in-hospital mortality compared to patients with EC who underwent laparoscopy (1.3% vs. 0.2%, *p* < 0.001). The share of postoperative prolonged mechanical ventilation was higher in the group of patients who underwent laparotomy compared to the group of patients who underwent laparoscopy (1.3% vs. 0.2%, *p* < 0.001). A total of 1551 patients (0.04%) undergoing laparoscopy were converted to laparotomy. Patients who underwent laparotomy had a longer hospital stay (13.7 ± 10.2 days vs. 7.2 ± 5.3 days, *p* < 0.001) and were more likely to be discharged toward another hospital or rehabilitation facility (*p* < 0.001) compared to patients who underwent laparoscopy (Table 2).

**Table 2:** In-hospital outcomes of the patients who underwent surgical therapy for endometrial cancer

	Laparotomy *n* = 45.639	Laparoscopy *n* = 38.793	Robotic-assisted laparoscopy *n* = 772	*p*-value Laparotomy vs. Laparoscopy	*p*-value Laparoscopy vs. Robotic-assisted laparoscopy
In-hospital mortality	1.3%	0.2%	0.00%	< 0.001	0.64
Ventilation > 48 h	1.3%	0.2%	< 0.4%*	< 0.001	0.70
Conversion to laparotomy	n/a	0.04%	0.00%	n/a	0.57
Length of stay (in days) ± SD	13.7 ± 10.2	7.2 ± 5.3	7.3 ± 5.9	< 0.001	0.84
Discharge home	95.2%	98.6	99.6%	< 0.001	0.01
Discharge 2^nd^ hospital	1.4%	0.5%	< 0.4%*	< 0.001	0.78
Discharge rehabilitation facility	0.8%	0.2%	< 0.4%*	< 0.001	0.66
Procedure costs in Euro (± SD)	8286 ± 7533	6047 ± 3509	7083 ± 3893	< 0.001	< 0.001

*SD* standard deviation

*Due to privacy concerns a share of < 0.4% is blinded by the federal bureau of statistics

### Outcomes of conventional laparoscopy and robotic-assisted laparoscopy for EC

None of the patients with EC staged by robotic-assisted laparoscopy died since the first recorded procedure in 2011 (Table 2). The proportion of patients with EC who required prolonged ventilation was similar in both treatment groups (0.2% laparoscopy vs. < 0.4% robotic-assisted laparoscopy, *p* = 0.70) (Table 2). No patient with EC who underwent robotic-assisted laparoscopy was converted to open surgery. The use of robotic-assisted surgery did not alter length of hospital stay compared to laparoscopic surgery (7.2 ± 5.3 days vs. 7.3 ± 5.9 days, *p* = 0.84). Patients undergoing robotic-assisted laparoscopy were more likely to be discharged home compared to patients undergoing laparoscopy (*p* = 0.01) (Table 2). Robotic-assisted laparoscopy is associated with higher procedure costs in comparison to laparoscopic surgery (robotic-assisted laparoscopy: 7083 ± 3893€; laparoscopy: 6047 ± 3509€, *p* < 0.001) (Table 2).

## Discussion

The present study analyzed the trends in surgical therapy and in-hospital outcomes of patients undergoing surgery for EC in Germany. Analysis of a nationwide dataset, including all cases who underwent surgery for EC between 2007 and 2018, revealed that minimal-invasive surgical procedures have increasingly become the standard surgical procedure for patients with EC. Furthermore, minimal-invasive staging surgery had superior in-hospital outcomes compared to laparotomy. Moreover, the use of robotic-assisted laparoscopy is increasing with comparable in-hospital safety profile to laparoscopy. This evidence is in line with data from other European countries [17].

EC is a major cause of morbidity and mortality, since it is a common cancer in women with rising incidence [18, 19]. Accordingly, we found increasing number of surgical procedures for EC during the analyzed period of time in Germany, possibly linked to an increasing incidence of the disease.

Although we found increasing use of robotic-assisted laparoscopy from 2011 until 2018, the share of robotic-assisted laparoscopies in all patients with EC in Germany was 3.2%. Compared to other countries, in Germany there is a long-lasting tradition in minimal-invasive techniques in gynecological surgery, especially in laparoscopic surgery [20]. This might be a reason for the relative slow increase in robotic-assisted laparoscopies for EC.

Two randomized trials showed the superiority of laparoscopic surgery with respect to in-hospital complications and quality of life compared to laparotomy for EC [21, 22]. The present study confirms these findings in real-world practice. Patients with EC who underwent minimally-invasive surgery had lower in-hospital mortality and were less frequently prolonged ventilated after surgery compared to patients who underwent laparotomy. A conversion to open surgery was a rare event and occurred in only 0.04% of all patients with EC who underwent laparoscopy. Moreover, the length of hospital stay was almost halved after minimal-invasive approach compared to laparotomy. These differences may occur, on one hand, a result of the surgical technique (laparotomy vs. minimally-invasive surgery). On the other hand, since the patients in the laparotomy group had a significantly higher CCI and the initial tumor stage was not available for the present analysis, there might be other reasons that lead to the indication of a specific surgical technique and thus lead to the described results.

Although robotic-assisted laparoscopy was less frequently used in the current study, in-hospital outcomes are comparable with conventional laparoscopic surgery for patients with EC. Until 2018 no reported death among patients with EC undergoing robotic-assisted surgery in Germany was recorded. Furthermore, no conversion from robotic-assisted laparoscopy to open surgery was necessary in accordance with available evidence [23, 24].

The nationwide introduction of robotic-assisted surgical staging in Denmark changed the surgical approach for early-stage EC from open surgery to minimal-invasive surgery [25]. This change in surgical approach was associated with a significantly reduced risk of severe complications [25]. Current evidence from the Danish Gynecological Database showed that the nationwide introduction of robotic-assisted laparoscopy for EC was associated with improved survival compared to open surgery [26]. This intriguing observation warrants further investigation.

The cost-effectiveness of robotic-assisted surgery is controversial and varies greatly in different surgical fields [27]. Our study suggests that robotic-assisted laparoscopy for EC is more cost effective compared to open surgery. However, a benefit in comparison to conventional laparoscopy has yet to be proven [28].

### Strengths and limitations

The strengths of this study include the analysis of a large nationwide dataset, with over 85 000 patients with EC, over a time span of 12 years. The dataset is well controlled by independent supervision. Therefore, it is reliable in respect to clinical characteristics and outcome risk analyses according to the three treatment groups. Several limitations need to be considered. Analyses were performed in a registry study setting from a national database according to ICD and OPS codes. Important clinical factors of patients with EC, e.g., tumor stage, initial extent of the disease, and subsequent adjuvant therapies, are missing in the present dataset precluding a balancing of the groups. Thus, the direct comparison between the three groups of patients has limitations, since higher tumor stage and disease extent at initial diagnosis determine the surgical technique. Furthermore, there was no follow-up available in order to evaluate long-term oncological outcomes of the three treatment groups.

The present analysis shows that minimal-invasive surgery has increasingly become the standard surgical technique for patients with EC in Germany, with excellent in-hospital outcomes in clinical practice. Robotic-assisted laparoscopy is an emerging surgical procedure in Germany with promising results compared to conventional laparoscopy.

## Data Availability

Not applicable.
